# Extinction of Conditioned Fear in Adolescents and Adults: A Human fMRI Study

**DOI:** 10.3389/fnhum.2017.00647

**Published:** 2018-01-08

**Authors:** Despina E. Ganella, Katherine D. Drummond, Eleni P. Ganella, Sarah Whittle, Jee Hyun Kim

**Affiliations:** ^1^Behavioral Neuroscience Division, The Florey Institute of Neuroscience and Mental Health, Parkville, VIC, Australia; ^2^Florey Department of Neuroscience and Mental Health, The University of Melbourne, Parkville, VIC, Australia; ^3^Melbourne Neuropsychiatry Centre, Department of Psychiatry, The University of Melbourne and Melbourne Health, Parkville, VIC, Australia

**Keywords:** adolescence, prefrontal cortex, memory, fear, behavior therapy

## Abstract

Little is known about the neural correlates of fear learning in adolescents, a population at increased risk for anxiety disorders. Healthy adolescents (mean age 16.26) and adults (mean age 29.85) completed a fear learning paradigm across two stages during functional magnetic resonance imaging (fMRI). Stage 1 involved conditioning and extinction, and stage 2 involved extinction recall, re-conditioning, followed by re-extinction. During extinction recall, we observed a higher skin conductance response to the CS+ relative to CS− in adolescents compared to adults, which was accompanied by a reduction in dorsolateral prefrontal cortex (dlPFC) activity. Relative to adults, adolescents also had significantly reduced activation in the ventromedial PFC, dlPFC, posterior cingulate cortex (PCC), and temporoparietal junction (TPJ) during extinction recall compared to late extinction. Age differences in PCC activation between late extinction and late conditioning were also observed. These results show for the first time that healthy adolescent humans show different behavioral responses, and dampened PFC activity during short-term extinction recall compared to healthy adults. We also identify the PCC and TPJ as novel regions that may be associated with impaired extinction in adolescents. Also, while adults showed significant correlations between differential SCR and BOLD activity in some brain regions during late extinction and recall, adolescents did not show any significant correlations. This study highlights adolescent-specific neural correlates of extinction, which may explain the peak in prevalence of anxiety disorders during adolescence.

## Introduction

Anxiety disorders have the highest lifetime prevalence of all mental disorders (Kessler et al., 2005) and adolescence has been identified as a vulnerable period for their emergence (Merikangas et al., 2011; Polanczyk et al., 2015). Rodent studies examining extinction of conditioned fear have aided our understanding of vulnerability to anxiety disorders during adolescence. Extinction of conditioned fear is the reduction in fear shown to a fear-inducing stimulus when it has been repeatedly presented without any threatening outcome. Notably, this process is thought to underlie vulnerability to anxiety disorders, and it is the most widely used model to understand exposure-based therapies (Milad and Quirk, 2012). We have shown that adolescent rats are impaired in extinction of a fear conditioned stimulus (CS) compared to adult rats (McCallum et al., 2010; Kim et al., 2011; Zbukvic et al., 2017). Specifically, adolescent and adult rats similarly acquire fear to a CS when it is paired with an unconditioned stimulus (US, e.g., footshock). The reduction in CS-elicited fear during extinction is also comparable. However, adolescent rats fail to remember extinction when tested the next day and show significantly higher levels of fear compared to older rats (i.e., adolescent rats show deficits in extinction recall), as well as compared to fear levels at the end of extinction (McCallum et al., 2010; Kim et al., 2011; Ganella et al., 2017; Zbukvic et al., 2017).

Given the relevance of extinction to anxiety vulnerability and treatment (Milad et al., 2009; Waters and Pine, 2016; Forcadell et al., 2017), it is imperative that we understand its underlying mechanisms. In human adults, extinction is consistently associated with ventromedial prefrontal cortex (vmPFC) activity (Phelps et al., 2004; Milad et al., 2005; Schiller et al., 2008; Hartley et al., 2011). Further, rodent studies present maturational changes in the vmPFC as a key mechanism underlying deficits in adolescent extinction at the protein, gene, and electrophysiological level (Kim et al., 2011; Pattwell et al., 2012; Zbukvic et al., 2017). In healthy humans, Pattwell et al. (2012) demonstrated that adolescents showed delayed within-session extinction compared to adults as measured by skin conductance response (SCR) to a visual CS+ paired with a shock compared to CS− that was never paired with a shock, although neural activity and extinction recall were not measured. In contrast, within-session extinction SCR in healthy youths and adults did not differ in another study (Britton et al., 2013). In that study, neural activity was not examined during within-session extinction, but functional magnetic resonance imaging (fMRI) was carried out during extinction recall several weeks later. During recall, threat appraisal was not modulated by age, and while vmPFC activity differed in clinically anxious youths and adults, healthy adolescents and adults did not show any differences in vmPFC activity during threat appraisal (Britton et al., 2013). However, SCR was not measured during extinction recall in that study, which poses difficulty in interpreting those findings in light of literature that predominantly uses SCR as the objective measure of fear learning (Milad and Quirk, 2012). Taken together, a substantial gap remains in our understanding of extinction deficits and underlying vmPFC activity in healthy human adolescents compared to adults.

Therefore, we investigated differences in the neural substrates of fear conditioning, extinction, and recall between adolescents and adults using fMRI while measuring SCR. We based the fear conditioning paradigm on that used by Britton et al. (2013), which involved the pairing of a neutral face (CS) with a fear face and scream (US) for the conditioning phase, which leads to robust subjective rating of anxiety to the CS in adolescents and adults (Britton et al., 2011, 2013; Lau et al., 2011). In addition, adolescents and adults show differences in habituation to a traditional shock US, therefore the fear face with scream is considered to be a better US (Lau et al., 2008). The CS+ was reinforced 100% with the US during conditioning based on other human fear trace conditioning studies (see Sehlmeyer et al., 2009; Fullana et al., 2015 for review). In particular, extinction of a CS that was previously reinforced 100% with the US has been predictive of individual differences in exposure therapy success in recent studies (Waters and Pine, 2016; Forcadell et al., 2017). Additionally, effective delineation of SCR and BOLD measures for CS vs. US in adolescence has been shown the with a CS that has been reinforced with the US 100% of the time (Cohn et al., 2016). The CS+ and CS− were presented without any outcome during extinction. After a short break, the CS+ and the CS− was presented once each to measure recall based on numerous studies have used one or two CS trials to measure extinction recall (Milad et al., 2005, 2006, 2007; Hartley et al., 2011; Rabinak et al., 2013; Forcadell et al., 2017). The recall trial for CS+ was followed by the same US as conditioning, however, it should be noted that the recall SCR and BOLD data were extracted before the US trial.

Our study is unique in that we have designed the study to investigate brain activity differences across phases within a single fMRI session. This is based on previous rodent work where the prominent dissociations in function of different brain regions are observed between the different stages of conditioning, extinction, and recall (Maren and Quirk, 2004; Hart et al., 2009; Ganella et al., 2017). Therefore, we predicted that age differences in brain activity would emerge when comparing different phases of our behavioral task. Our primary hypothesis was that adolescents, compared to adults, would show reduced activity in the vmPFC during extinction recall compared to the end of extinction. Based on prior research examining the neural systems of conditioning and extinction in both humans and rodents, we additionally hypothesized that there would be age-related differences in activation in other core regions of the fear learning network; the amygdala, hippocampus, dorsolateral PFC (dlPFC), dorsomedial PFC (dmPFC) and insula (LaBar and LeDoux, 1998; Phelps et al., 2004; Milad et al., 2005; Hartley et al., 2011; Kim et al., 2011).

## Materials and methods

### Participants

All participants (and their parents if <18 years of age) provided written informed consent to participate in the study, which was approved by the Royal Children's Hospital Research Ethics Committee: 34141A, Victoria, Australia. In total 18 adult participants (aged 25–35) were recruited from the community and 20 adolescents (aged 14–16) were recruited from schools in Melbourne, Australia. Exclusion criteria included (i) current treatment for a psychiatric illness, (ii) non-native English speaker, (iii) current psychoactive medication use, (iv) pregnant, and (v) contraindications to MRI. Data from 14 adults (6 females, M age 29.85 years, S.D. 3.03 years) and 17 adolescents (10 females, M age 16.26 years, S.D. 0.4 years) were included in analyses after exclusions based on technical scanner issues (*n* = 1 adult), image acquisition problems (*n* = 1 adult) and excessive head motion (*n* = 2 adults, *n* = 3 adolescents).

### Magnetic resonance imaging assessment–image acquisition parameters

Neuroimaging data were acquired on a 3T Siemens TIM Trio scanner (Siemans, Erlangen, Germany) at the Murdoch Childrens Research Institute, Royal Children's Hospital, Melbourne, Australia. Participants lay supine with their head supported in a 32-channel head coil and headphones. For stage one of the task (Conditioning and Extinction), 296 whole-brain T2^*^-weighted echo-planar images [repetition time (TR) = 3,000 ms, echo time (TE) = 40 ms, pulse angle = 85°, field of view (FOV) = 216 mm] were acquired, corresponding to 40 interleaved slices with a voxel size of 3 × 3 × 3 mm. For stage two of the task (Recall, Re-conditioning and Re-extinction), 170 whole-brain T2^*^-weighted echo-planar images with the same parameters as stage one were acquired. T1-weighted MPRAGE images were also acquired for co-registration purposes (TR = 2,530 ms, TE = 1.74 ms, flip angle = 7°, FOV = 256 × 208 mm), producing 176 1 mm contiguous sagittal slices (voxel dimensions = 1 mm^3^).

### Procedure

**Figure 1:**
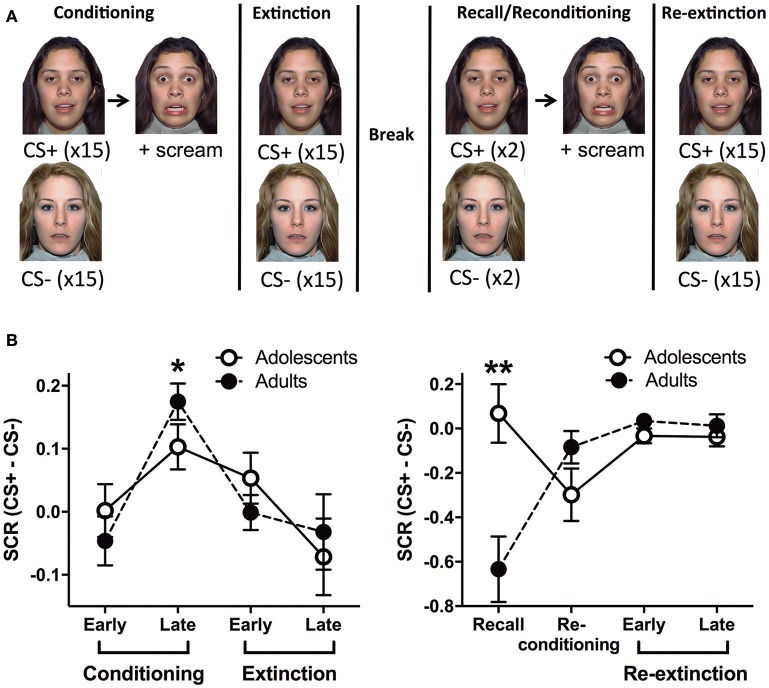
Outline of the behavioral paradigm and corresponding skin conductance response data. **(A)** A schema of the behavioral paradigm: conditioning, extinction, recall, re-conditioning and re-extinction. Face stimuli are from NimStim set of facial expressions http://www.macbrain.org/resources.htm with the permission to publish these stimuli. **(B)** Skin conductance response (SCR) for adults and adolescents during the functional magnetic resonance imaging paradigm (fMRI). Stage 1 - conditioning and extinction; late conditioning was significantly different to early conditioning and late extinction (^*^ps < 0.05). Stage 2 - recall, re-conditioning and re-extinction; there was a significant age effect with adolescents showing more SCR compared to adults (^**^p < 0.0001). CS, conditioned stimulus.

The task was presented with Paradigm software (http://www.paradigmexperiments.com), running on a Dell computer. The LCD screen that presented stimuli was visible via a reverse mirror mounted to the participants' head coil. Familiarization of the task was conducted outside the scanner, where participants were presented with two neutral female faces taken from the NimStim set of facial expressions http://www.macbrain.org/resources.htm with permission to publish stimuli used in Figure 1 in the present study (Tottenham et al., 2009). These faces were different from the CS+ and CS− faces presented in the scanner. Skin conductance response (SCR) was collected using a galvanic skin response amplifier (ADInstruments, Milford, MA) with two electrodes attached with a Velcro strap (and electrolyte gel) to the palmar surfaces of the index and middle fingers of the non-dominant hand. LabChart (ADInstruments, Milford, MA) was used to extract the SCR data (digital low pass filter and cut-off frequency 0.1 Hz). It is important to note that this equipment measures relative conductance value from the beginning of experimentation rather than absolute value, therefore the raw values can be positive or negative.

### Event related paradigm

Adults and adolescents received the same behavioral paradigm with two stages whilst undergoing a fMRI scan. Stage 1: Conditioning, where one of two neutral faces (CS+) was presented for 3 s followed by 2–4 s jittered trace period then a fear face (1 s) and female scream (US, ~95–100 dB). The jittered interval was chosen based on Lau et al. (2011) and Britton et al. (2013) for optimal fMRI analyses and to reduce US habituation (Lake et al., 2016). The CS+ was reinforced 100% with the US during conditioning based on other human fear trace conditioning studies, see (Sehlmeyer et al., 2009; Fullana et al., 2015) for review. In particular, extinction of a CS that was previously reinforced 100% with the US has been predictive of individual differences in exposure therapy success in recent studies (Waters and Pine, 2016; Forcadell et al., 2017). The other face (CS−, 3s) was used as a control stimulus that was never paired with the US. CS+US trials and CS− trials were interleaved and were presented 15 times each in random order. The inter-trial interval (ITI) was a white fixation cross on a black background, jittered for 8–12 s. Extinction followed immediately, 15 CS+ (3 s) trials in the absence of the US, randomly interleaved with 15 CS− (3 s) trials. After extinction there was a ~10 min rest prior to recall, based on similar timing used in previous studies (LaBar and Phelps, 2005; Den et al., 2015). Stage 2: Recall trials were given, in which the CS+ and the CS− was presented once each to measure extinction recall (Milad et al., 2005, 2006, 2007; Hartley et al., 2011; Rabinak et al., 2013; Forcadell et al., 2017). The recall trial for CS+ was followed by the same US as conditioning, however, it should be noted that the recall SCR and BOLD data were extracted before the US trial. There was then a trial each of CS+US and CS−. Because these CSs were presented after the previous US, we name this trial “Re-conditioning.” These trials were followed by a Re-extinction phase, which was identical to the Extinction phase. The two faces were counterbalanced as CS+ or CS− and there were no more than 2 consecutive trials of the CS+ or CS−.

### Statistical analyses

SCR was collected throughout the paradigm, and extracted using ADInstruments Labchart software. SCRs to each CS+ and CS− were identified by the peak skin conductance level within the 5 seconds from CS onset (Pattwell et al., 2012). Across all the phases, the peak SCR value for each CS+ or CS− was never confounded with the US trial that always began 5+ seconds from CS onset. These peak values were then averaged across blocks of 5 CS+ and 5 CS− for each phase of the paradigm, except for recall and re-conditioning phase (the first CS+ trial before the US was presented was named “recall” (Forcadell et al., 2017), and the second CS+US trial was named “re-conditioning”). Analyses were then carried out on difference scores (CS+ minus CS−, Pattwell et al., 2012), and data are represented as such (± standard error of the mean). Repeated-measures Analyses of Variance (ANOVA) was used to assess changes in SCR across different phases for each Stage, with Age (adolescent vs. adult) as the between-subjects factor. Significant main effects were followed up with Tukey honest significant difference (HSD) *post-hoc* multiple comparisons, and significant interactions were followed up with Bonferroni *t*-tests for each phase with Age as the between-subjects factor.

For fMRI, four “dummy” volumes acquired at the beginning of each stage were discarded to allow for T1 equilibration effects. We performed pre-processing procedures using Statistical Parametric Mapping (SPM) 12 (http://www.fil.ion.ucl.ac.uk/spm/). These included slice timing correction, motion correction, co-registration of functional images with participants' T1-weighted image, which had been co-registered to the SPM-T1 template. Co-registered volumes were concurrently re-sliced to 2 mm isotropic resolution and normalized to SPM-T1 template. The resulting transformation matrix was applied to the functional data to achieve accurate spatial normalization across individuals. Finally, functional images were smoothed using a Gaussian filter (full-width at half maximum, 6 mm). Head motion was inspected for each participant; maximal amplitude of translational and rotational displacements (x, y, z) were required to be <3 mm or 3°, respectively for all participants as reported in other studies (Cignetti et al., 2016; Ginther et al., 2016). Those which exceeded this threshold were excluded from analysis as reported in other studies (Cignetti et al., 2016; Ginther et al., 2016).

The time series data were subjected to a general linear model, convolved with the canonical hemodynamic response function (HRF) and filtered with a 128s high-pass filter. We modeled CS+, CS−, trace period, US and rest periods separately and estimated effects for each voxel for each participant. Individual motion parameters were entered in the model as covariates of no interest. Following processing at the first-level, contrasts of interest were taken to the second level for random effects analysis. As per previous literature (Phelps et al., 2004; Milad et al., 2007), early (first 5 CS+ and first 5 CS− trials) and late (last 5 CS+ and last CS− trials) stages of conditioning, extinction and re-extinction phases were modeled in contrasts. Whole-group (across adults and adolescents) and between-group “Age” (adults>adolescents; adolescents>adults) comparisons were performed for each contrast of interest (Tables 1, 2). Within-group (adults and adolescents) effects were also investigated for completeness. Results for additional across phase contrasts, within-phase contrasts (e.g., early vs. late extinction) and simple contrasts (e.g., early extinction CS+ > CS−) are provided in Table 3.

**Table 1:** Contrasts of interest with rationales.

**Contrast of Interest**	**Rationale**
Recall	The first CS presentation following rest after extinction assesses how well the extinction memory is retrieved—consistent age differences in extinction is observed at this phase in previous rodent studies.
Recall vs. late extinction	Late extinction represents when extinction learning is more complete and the CS-no US outcome is no longer surprising. Comparing this to the recall phase shows whether brain regions are differentially involved when extinction is learnt compared to when extinction memory is retrieved.
Early extinction vs. early conditioning	The early trials of extinction and the early trials of conditioning are when the outcome following the CS is the most surprising (i.e., largest error correction is occurring). Comparing these two phases will identify whether brain regions are differentially engaged when the learning is the greatest but occurring in opposite directions (CS-US vs. CS-no US).
Late extinction vs. late conditioning	Comparing these two phases show whether different brain regions are engaged at later phases of learning when the CS predict opposite outcomes, however, the outcomes are no longer surprising.
Early extinction vs. early re-extinction	While these phases are identical in procedure, rodent literature suggests that extinction and re-extinction involve distinct brain regions because while extinction is a new learning, re-extinction is hypothesized to be a retrieval of the extinction memory. However, this idea has never been tested in adolescence. Comparing these two phases will reveal any age differences in transition from extinction to re-extinction.
Late extinction vs. late re-extinction	Again, while these phases are identical in procedure, given that different brain regions have been shown to be necessary for extinction and re-extinction, examining this contrast will show whether the same brain region is differentially involved in the different phases. Age related differences in these phases have not been previously examined.

**Table 2:** fMRI results for contrasts of interest.

**Contrasts of Interest**	**Voxels (n)**	***t***	**MNI coordinates**		
			***x***	***y***	***z***
**Recall (CS+ > CS−)**					
*Adults* > *Adolescents*					
R dlPFC	153	4.33	36	44	10
R PCC^*^	599	4.89	8	−50	38
*Adults*					
R Superior parietal cortex	932	4.83	26	−30	66
**Recall (CS+ > CS−) vs. Late Extinction (CS+ > CS−)**					
*Adults* > *Adolescents*					
R vmPFC^*^	364	4.76	14	48	−6
R dlPFC/frontal pole	172	3.45	36	44	10
R PCC^*^	3,757	5.93	8	−50	38
R TPJ	587	4.74	42	−56	24
*Adults*					
L dlPFC/frontal pole^*^	225	4.13	−24	46	32
L rACC	150	3.39	−22	50	2
R Precentral gyrus	708	4.71	12	−16	54
**Early Extinction (CS+ > CS−) vs. Early Conditioning (CS+ > CS−)**					
*Adolescents* > *Adults*					
R vmPFC	175	4.61	12	38	−4
*Adolescents*					
R vmPFC^*^	683	5.92	12	38	−4
*Whole group effect*					
R vmPFC	487	4.97	14	42	−2
**Late Extinction (CS+>CS−) vs. Late Conditioning**					
*Adults* > *Adolescents*					
L PCC^*^	652	5,345	−8	−52	28
*Adults*					
L PCC^*^	903	4.27	−4	−56	32
**Early Re-Extinction (CS+ > CS−) vs. Early Extinction (CS+ > CS−)**					
*No significant findings*					
**Late Extinction (CS+ > CS−) vs. Late Re-extinction (CS+ > CS−)**					
*No significant findings*					

All results survived cluster correction p < 0.05 using a cluster forming threshold of p < 0.005. Those surviving a cluster forming threshold of p < 0.001 are indicated by^*^.

*R, right; L, left; vmPFC, ventromedial prefrontal cortex; PCC, posterior cingulate cortex; TPJ, temporoparietal junction; dlPFC, dorsolateral prefrontal cortex; rACC, rostral anterior cingulate cortex*.

**Table 3:** fMRI results for within-condition and simple contrasts.

**Within-condition Contrasts**	**Voxels (*n*)**	***t***	**MNI coordinates**		
			***x***	***y***	***z***
**Recall (CS+ > CS−) vs. Early** **Re-extinction (CS+ > CS−)**					
*Adults > Adolescents*					
R PCC	691	5.13	12	−44	34
**Early Re-extinction (CS+ > CS−) vs.** **Late Extinction (CS+ > CS−)**					
*Adults*					
L Mid/PCC^*^	896	5.96	−10	−14	50
R Lateral Occipital	847	5.28	24	−78	22
**Late Conditioning (CS+ > CS−) vs.** **Early Extinction (CS+ > CS−)**					
*No significant findings*					
**Early Re-extinction (CS+ > CS−) vs.** **Late Re-extinction (CS+ >CS−)**					
*No significant findings*					
**Late Conditioning (CS+ > CS−) vs.** **Early Conditioning**					
*Whole group effect*					
R TPJ^*^	664	4.72	56	−48	24
L Precuneus	804	4.56	−10	−78	46
L PCC	521	4.55	−8	−28	36
R Supramarginal gyrus	554	4.23	48	−36	58
R dmPFC/rACC	233	4.04	10	50	26
**Late Extinction (CS+ > CS−) vs.** **Early Extinction (CS+ > CS−)**					
*No significant findings*					
**Early Conditioning (CS+ > CS−) vs.** **Mid Conditioning (CS+ > CS−)**					
*Whole group effect*					
L PCC	501	3.95	−14	−38	42
**Simple Contrasts**	**Voxels (*****n*****)**	***t***	***x***	***y***	***z***
**Early Conditioning (CS+ > CS−)**					
*Whole group effect*					
L Precuneus	1,252	4.20	−4	−50	64
**Late Conditioning (CS+ > CS−)**					
*Adolescents*					
R dlPFC/middle frontal gyrus^*^	541	5.13	48	20	28
**Early Extinction (CS+ > CS−)**					
*No significant findings*					
**Late Extinction (CS+ > CS−)**					
*Adolescents > Adults*					
R rACC/dmPFC^*^	302	4.60	16	46	20
**Early Re-extinction (CS+ > CS−)**					
*No significant findings*					
**Late Re-extinction (CS+ > CS−)**					
*Adolescents*					
R vmPFC	214	3.89	8	46	−8
**Re-conditioning (CS+ > CS−)**					
*Adults > Adolescents*					
L PCC	534	4.30	−10	−52	36

All results survived cluster correction p < 0.05 using a cluster forming threshold of p < 0.005. Those surviving a cluster forming threshold of p < 0.001 are indicated by ^*^.

*R, right; L, left; vmPFC, ventromedial prefrontal cortex; PCC, posterior cingulate cortex; dmPFC, dorsomedial prefrontal cortex; dlPFC, dorsolateral prefrontal cortex; rACC, rostral anterior cingulate cortex*.

Second-level results were corrected for multiple analyses using a cluster-level threshold of *p* < 0.05 family-wise error (FWE) correction as determined by AFNI's 3dClustSim program (version 16.3.09) using 20,000 iterations, a mask of the whole brain, and a smoothness estimated using 3dFWHMx with the –ACF option. Given priori hypotheses about the role of the amygdala, vmPFC, hippocampus, dmPFC, dlPFC, and insula, a bilateral mask of these regions was created using the WFUpickatlas toolbox. Clusters within this combined region of interest (ROI) were thresholded to achieve a small volume corrected *p* = 0.05, as determined by 3dClustSim (using the same parameters as described above). For all analyses, results were considered significant using a cluster forming threshold of *p* < 0.005 (results surviving a threshold of *p* < 0.001 are noted in Table 1). For contrasts where there was a significant effect of age, BOLD signal from a 6 mm sphere from the peak coordinates of the significant cluster were extracted from each condition comprising the effect for each participant for plotting, post-hoc Bonferroni paired *t*-tests to compare CS+ and CS− within each phase in each age group, and for investigating associations with SCR.

## Results

### Physiological data–skin conductance response

In stage 1 (conditioning and extinction), repeated measures (RM) analysis of variance (ANOVA) showed a significant effect of Block [*F*_(3, 87)_ = 6.282, *p* = 0.0007]. Tukey HSD multiple comparisons revealed that late conditioning was significantly different to early conditioning and late extinction (^*^p's < 0.05) (Figure 1B). This indicates that SCR for CS+ compared to CS− significantly increased by late conditioning compared to early conditioning, and then it significantly decreased by late extinction. We found no effects of Age or Age × Block interaction (smallest *p* = 0.45). In stage 2 (recall, re-conditioning and re-extinction), RM ANOVA showed a significant Age × Block interaction [*F*_(3, 87)_ = 8.842, *p* < 0.0001]. Due to the significant interaction, we analyzed each Block separately, which revealed that there was a significant Age effect for the recall trial with adolescents showing more SCR compared to adults (^**^p < 0.0001; Figure 1B). There were no effects at any other block. There was also a significant effect of Block [*F*_(3, 87)_ = 3.987, *p* = 0.0103], however, Tukey HSD multiple comparisons indicated no effects between pairs of blocks (smallest *p* = 0.064). There was no effect of Age (*p* = 0.054).

### fMRI

#### Recall

**Figure 2:**
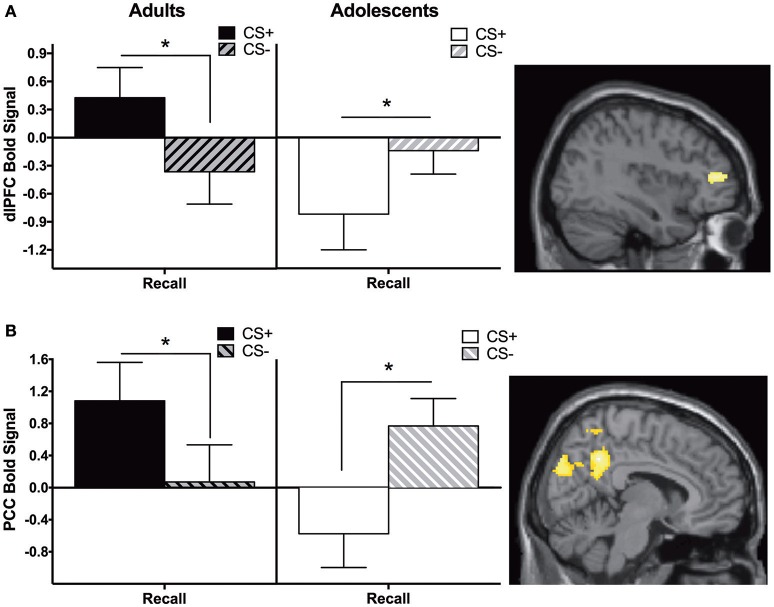
Recall contrast. Adults showed significantly greater activation in the **(A)** dorsolateral prefrontal cortex (dlPFC) and **(B)** posterior cingulate cortex (PCC) compared to adolescents during the recall phase (CS+ > CS−). *Post-hoc* tests identified significant differences between CS+ and CS− at each phase for each age in dlPFC and PCC (^*^ps < 0.05). Graph generated from BOLD signal from a 6 mm sphere around the peak coordinates of the significant cluster.

Adults showed significantly greater CS+ > CS− activity in the dlPFC and posterior cingulate cortex (PCC) compared to adolescents during recall (Figure 2, Table 2).

#### Recall vs. late extinction

**Figure 3:**
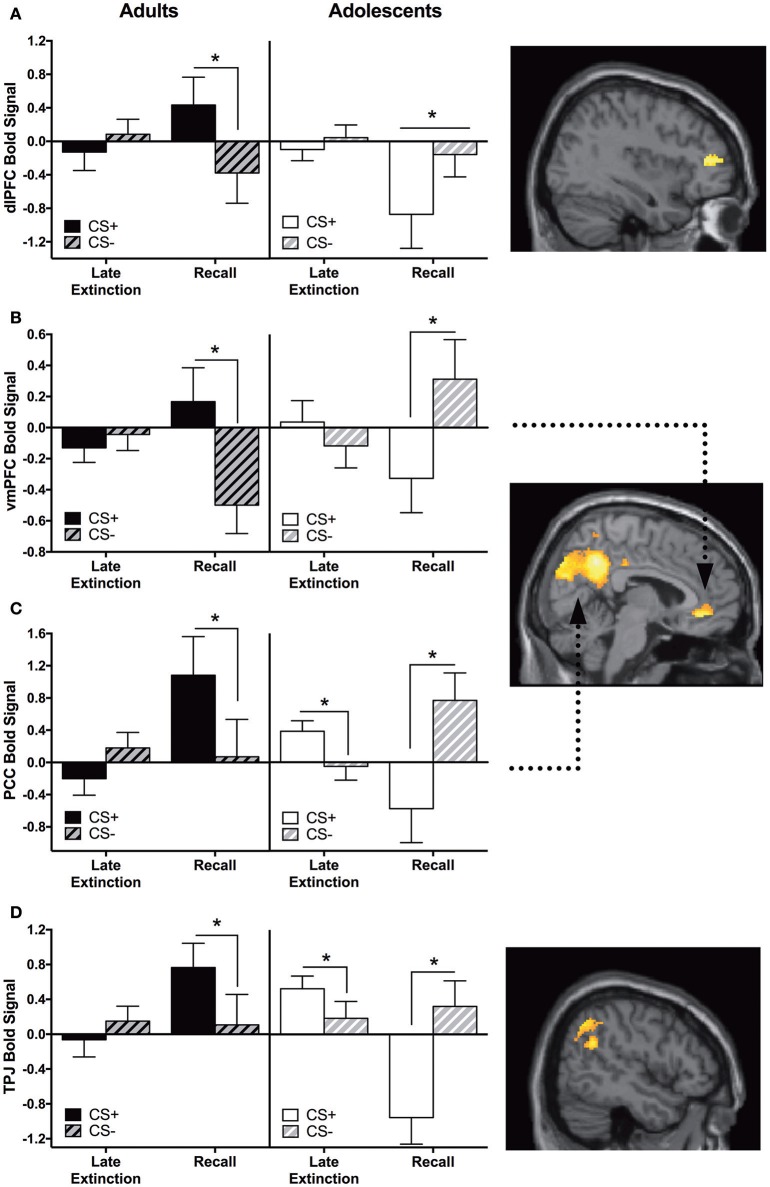
Recall vs. late extinction contrast. Adults showed significantly greater activation in the **(A)** dorsolateral prefrontal cortex (dlPFC), **(B)** ventromedial prefrontal cortex (vmPFC), **(C)** posterior cingulate cortex (PCC), and **(D)** temporoparietal junction (TPJ) compared to adolescents during the recall phase (CS+ > CS−) vs. late extinction (CS+ > CS−). In these brain regions, *post-hoc* tests identified significant differences between CS+ and CS− within recall phase in each age group (^*^ps < 0.05). In PCC and TPJ, CS+ and CS− activation was also significantly different during late extinction for adolescents (^*^ps < 0.05). Graph generated from BOLD signal from a 6 mm sphere around the peak coordinates of the significant cluster. Arrows highlight the vmPFC and PCC regions.

Adults showed significantly greater CS+ > CS− activation in the vmPFC, dlPFC, PCC, and temporoparietal junction (TPJ) compared to adolescents during recall vs. late extinction (Table 2). The pattern of activation for CS+ and CS− appeared to be reversed for adults compared to adolescents for each region, with the age difference driven by greater CS+ relative to CS− activation in adults compared to adolescents for extinction recall *(ps* < 0.05) (Figures 3A,B). The age effect in the PCC and the TPJ was also driven by adolescents showing significantly higher CS+ activation relative to CS− during late extinction (Figures 3C,D).

#### Early extinction vs. early conditioning

**Figure 4:**
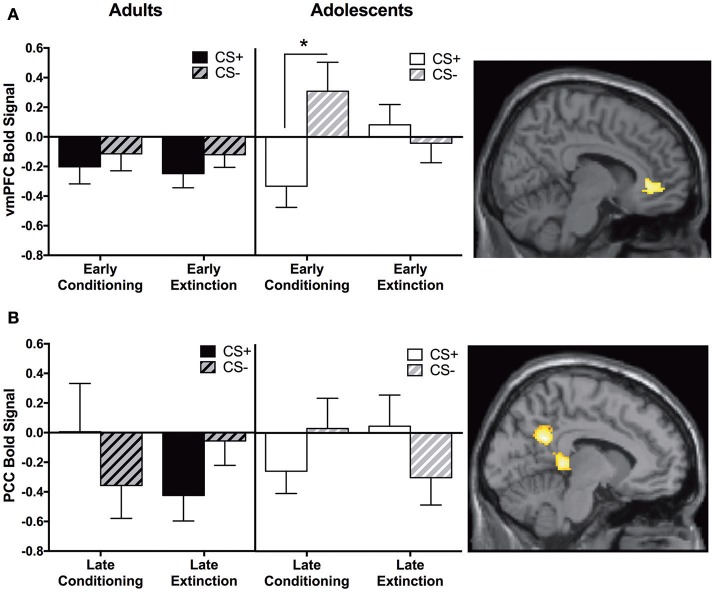
Early extinction vs. early conditioning contrast and late conditioning vs. late extinction contrast findings. **(A)** Early extinction vs. early conditioning contrast. Adolescents showed significantly greater activation in the ventromedial prefrontal cortex (vmPFC) compared to adults during early extinction (CS+ > CS−) vs. early conditioning (CS+ > CS−). *Post-hoc* tests identified a significant difference between CS+ and CS−, in adolescents during early conditioning (^*^p < 0.05). **(B)** Late conditioning vs. late extinction contrast. Adolescents showed significantly greater activation in the posterior cingulate cortex (PCC) compared to adults during late extinction (CS+ > CS−) compared to late conditioning (CS+ > CS−). Graphs generated from BOLD signal from a 6 mm sphere around the peak coordinates of the significant cluster.

We identified a main effect for the whole group in the vmPFC for this contrast; however, this was moderated by Age. Adolescents showed greater CS+ > CS− activity in the vmPFC during early extinction vs. early conditioning, and this activation was significantly different compared to adults. Post-hoc tests revealed that adolescents showed greater CS− relative to CS+ activation during early conditioning (*p* < 0.05, Figure 4A, Table 2).

#### Late extinction vs. late conditioning

Adults showed significantly greater CS+ > CS− activation in the PCC compared to adolescents during late conditioning vs. late extinction (Table 2). This pattern of activation was reversed in adolescents (Figure 4B). *Post-hoc* paired *t*-tests were not significant for any phase at any age (*ps* = 0.1–0.14).

#### Correlation analyses between SCR and brain activity

**Figure 5:**
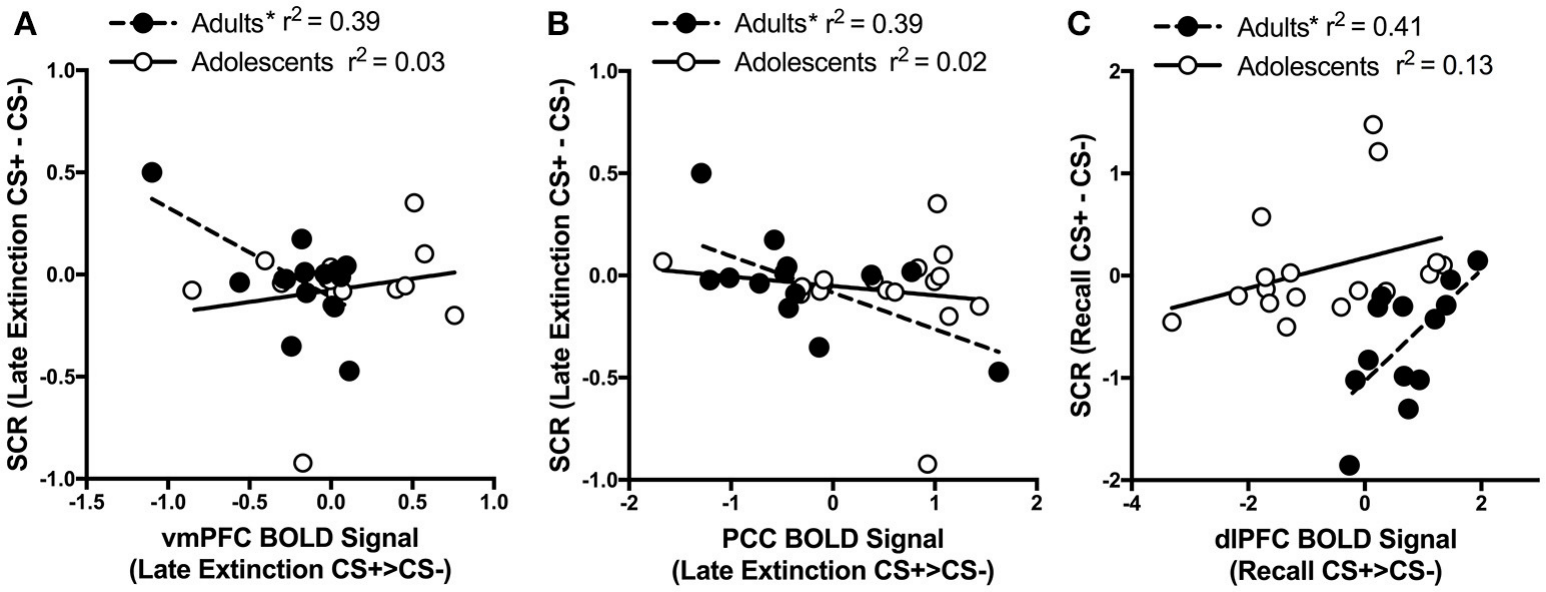
Correlations between brain activity and skin conductance response. **(A)** During late extinction, ventromedial PFC (vmPFC) activity negatively correlates with skin condutance response (SCR) in adult but not in adolescent participants (Adults *r* = −0.63; Adolescents *r* = 0.19). **(B)** Posterior cingulate cortex (PCC) activity during late extinction negatively correlates with SCR in adult but not in adolescent participants (Adults *r* = −0.62; Adolescents *r* = −0.144). **(C)** Dorsolateral prefrontal cortex (dlPFC) activity during recall positively correlates with SCR during recall in adult but not in adolescent participants (Adults *r* = 0.64; Adolescents *r* = 0.36). ^*^Indicates a significant correlation *p* < 0.05.

For adults and adolescents separately, we correlated BOLD signal (CS+ > CS−) with differential SCR during each experimental phase for each brain region where significant age differences were found. Three significant findings emerged. During Late Extinction, there was a significant negative correlation in adults between SCR and brain activity in both the vmPFC (adults; *r* = −0.63, *p* = 0.02, adolescents; *r* = 0.19, *p* = 0.48) and PCC (adults; *r* = −0.62, *p* = 0.02, adolescents; r = −0.144, *p* = 0.58) (Figures 5A,B). There was a significant positive correlation between SCR and dlPFC activity during recall for adults (*r* = 0.64, *p* = 0.02), but not in adolescents (*r* = 0.36, *p* = 0.15) (Figure 5C).

## Discussion

In the present study, we observed age differences in the behavioral and neural correlates of fear extinction. Adolescents showed significantly higher SCR and reduced activation in the PCC and the dlPFC compared to adults during extinction recall. Adolescents also showed reduced vmPFC, dlPFC, TPJ, and PCC activation compared to adults during recall relative to late extinction. Interestingly, adolescents compared to adults showed increased vmPFC activation during early extinction relative to early conditioning, and increased PCC activity during late extinction relative to late conditioning despite similar SCR in adolescents and adults during these phases. While adults showed significant correlations between differential SCR and BOLD activity in some brain regions during late extinction and recall, adolescents did not show any significant correlations.

### Short-term extinction recall differences between adolescents and adults (SCR)

We observed significantly higher SCR to CS+ vs. CS− in adolescents relative to adults during extinction recall, with no other age associated SCR differences throughout the paradigm, a finding consistent with previous rodent studies (McCallum et al., 2010; Kim et al., 2011; Zbukvic et al., 2017). Of note, the age difference in differential SCR appeared to be driven by higher SCR to CS− vs. CS+ in adults (i.e., negative average differential score in adults), although there were no age differences in CS+ or CS− separately (i.e., the significant age differences was only for the differential SCR score). Further, adults did appear to show elevation in SCR following re-conditioning, suggesting that extinction recall was successfully reversed in this age group. Adolescents did not appear to show further increases in SCR following re-conditioning, which might suggest that they did not recall extinction in the first place. Our findings show for the first time that human adolescents show different behavioral responses during extinction recall compared to adults, at least when short-term recall is assessed. However it's important to note that the interpretation of age differences in SCR during recall is not straight forward, and the current lack of prior research makes it difficult to compare our findings. For example, while Pattwell et al. (2012) observed impaired within-session extinction learning in adolescent humans, with children and adults showing a greater reduction of SCR between the first two trials of extinction and the last two trials of extinction compared to adolescents, extinction recall was not assessed, The differences we observe may indicate an adolescent extinction deficit (consistent with other research showing, for example, that adolescent rats display impairments in extinction of a drug-associated cue and context, Brenhouse and Andersen, 2008; Zbukvic et al., 2016), although further research is needed to corroborate this interpretation.

It is interesting to note that we did not observe SCR differences between adolescents and adults during conditioning, which is consistent with previous studies (Lau et al., 2011; Pattwell et al., 2012; Britton et al., 2013). Lau et al. (2011), Britton et al. (2013), and Pattwell et al. (2012) used delayed conditioning in which CS+ and the US co-terminated. These three studies used partial reinforcement of the CS+. Thus, overall, healthy adolescents and adults appear to show comparable threat conditioning, at least as measured by SCR. These findings strengthen the idea that the two age groups differ in extinction rather than fear conditioning.

### PFC activity in adolescents compared to adults

We observed reduced adolescent vmPFC activity during extinction recall, along with reduced activity in the dlPFC relative to adults. The involvement of the vmPFC in extinction recall is consistent with previous human research. For example, vmPFC structure and activation is associated with successful extinction recall in adult humans (Phelps et al., 2004; Milad et al., 2005, 2007, 2009). Our finding of reduced vmPFC activity in adolescents may suggest an underlying neural mechanism for adolescent impairments in recalling extinction, which is consistent with previous findings in rodents (Kim et al., 2011; Pattwell et al., 2012).

While the reduced vmPFC activation in adolescents during recall compared to late extinction was mainly driven by the recall phase, findings also suggest that adolescents may engage the vmPFC more than adults during late extinction. Interestingly, enhanced vmPFC activity to CS+ and CS− reversal learning has been observed in adults (Schiller et al., 2008). This finding together with our results suggest that enhanced vmPFC during late extinction in adolescents may indicate extinction being experienced as a reversal learning at this age. That is, rather than learning that both CS+ and CS− no longer signal the US, adolescents may expect the CS− to acquire the predictive properties of CS+. Indeed, Norrholm and colleagues discuss reversal learning as a unique feature of discrimination conditioning in human fear extinction (Norrholm et al., 2006, 2008). In support, in the present study, adolescents showed *greater* vmPFC activity to the CS+ > CS− during early extinction vs early conditioning (Figure 4A). We hypothesize that the capacity to discriminate “threat” from “safety” during conditioning is unrefined at this age, which may contribute to development of anxiety disorders (Britton et al., 2011). Our correlational data are consistent with this idea, with adult vmPFC activity for the CS+ compared to CS− negatively correlating with the magnitude of CS+ compared to CS− SCR during late extinction (Figure 5). No such effect was observed in adolescents. This suggests that the adult brain may be discriminately processing the CS+ and the CS− by the end of extinction with more activity in the vmPFC leading to less SCR to the CS+ compared to CS− whereas adolescents are not. In fact, clinically anxious adolescents showed a markedly smaller difference in their conditioned fear to the CS+ vs. CS− than adults using both subjective responding and SCR, and this was associated with age differences in brain activity (Lau et al., 2011). Our results suggest that adolescents may be incorrectly appraising the initial threat during early conditioning, which leads to impairments in early extinction and may continue into the late phase of extinction.

The reduced dlPFC activation to the CS+ in adolescents compared to adults during extinction recall vs. late extinction is interesting because the dlPFC underlies higher cognitive functions such as executive processing and working memory (Smith and Jonides, 1999; Delgado et al., 2008). Recently, the dlPFC has been identified to be crucial in voluntary forgetting, with transcranial magnetic stimulation of the dlPFC enhancing voluntary forgetting (Hanslmayr et al., 2012). Therefore, adults may be able to intentionally forget the original CS-US association and retrieve the extinction memory better than adolescents due to increased dlPFC function. Additionally, increased activity in the dlPFC during emotion suppression has been correlated with decreased intensity of experienced negative emotion (Phan et al., 2005). Our results may thus suggest that adolescents are not down-regulating the negative emotions associated with CS+ as effectively as adults. However, our correlational data show that increased CS+ > CS− activity was correlated with increased CS+ vs. CS− SCR during recall in adults. This suggests that the role of the dlPFC during extinction recall in adolescents may be more cognitive rather than reflective of suppression of emotions. It has been proposed that cognitive emotion regulation strategies that recruit dlPFC regions may diminish emotional responses through connection with vmPFC (Delgado et al., 2008). Given that we observed reduced activity in both the dlPFC and vmPFC during recall in adolescents, there may be a compounded effect contributing to the extinction recall deficit. Taken together, these findings indicate that these prefrontal brain regions are central to adolescent deficits in extinction recall.

### PCC and TPJ as novel loci underlying extinction differences between adolescents and adults

Our findings of age differences in PCC and TPJ involvement in extinction were unexpected, and highlight these regions as novel targets for future investigation. Specifically, adults showed greater activation in the PCC (CS+ > CS−) compared to adolescents during extinction recall, whereas adolescents showed greater PCC activation during late extinction. The PCC has a well-established role in the default mode network (DMN), a system of brain regions more active at “rest” compared to during a cognitively demanding task (Fransson and Marrelec, 2008). More recently, the PCC has also been ascribed roles in episodic memory retrieval and responding to behaviorally relevant emotional stimuli (Maddock et al., 2002; Kim, 2010). If the PCC is working as part of the DMN, the lack of adolescent PCC deactivation to the CS+ during late extinction may suggest that adolescents are not as cognitively engaged, which may lead to reduced learning to the CS+ relative to CS− during extinction. In support of this idea, PCC activation to the CS+ during late extinction was not correlated with SCR in adolescents, whereas in adults higher PCC activation was related to decreased SCR. If PCC activation is interpreted in terms of engagement in episodic memory retrieval, adolescent PCC activation to the CS+ during late extinction may reflect an ongoing effort to retrieve safety information about the CS+. PCC deactivation to the CS+ during recall may reflect failure to retrieve safety information about the CS+. There is accumulating evidence for PCC dysfunction underlying many childhood/adolescent-onset mental disorders (Leech and Sharp, 2013). Further, anxiety disorder patients show an association between increased extinction–related PCC activity and greater symptom severity (Milad et al., 2013). Our findings suggest that PCC dysfunction may also play a role in anxiety disorders during adolescence.

Lastly, reduced activity in the TPJ was found in adolescents compared to adults during recall vs. late extinction. The TPJ is activated when attention is captured by behaviorally relevant stimuli (Serences et al., 2005), and tends to deactivate during cognitively demanding tasks such as working memory (Anticevic et al., 2010; Schott et al., 2011). During recall, the TPJ was deactivated to the CS+ in adolescents, whereas adults show activation to the CS+ during this phase. Therefore, our results suggest that adolescents may require stronger engagement of their working memory to recall extinction and/or are misattributing what is behaviorally relevant compared to adults.

### Strengths, weaknesses, and future directions

Although our study's sample size is larger or comparable to previous studies of this kind (Phelps et al., 2004; Milad et al., 2005, 2009; Schiller et al., 2008; Lau et al., 2011; Rabinak et al., 2013), it was not large enough to examine gender differences, which should be done in future work given that females are twice as likely to suffer from an anxiety disorders than males. Strengths of our study include the usage of SCR as an objective measure of fear learning and extinction, as well as thorough within-and cross-phase comparisons of SCR and BOLD measurements. While the interpretation of age differences in SCR during recall was not straight forward (i.e., results suggested greater responding to the CS− in adults), we only went so far as to probe SCR effects, given that this was not the main focus of our study. It is important to note, however, that neural changes are commonly observed in the absence of physiological changes (e.g., SCR), which highlights a particular sensitivity of fMRI in detecting potential dysfunctions (Rougemont-Bücking et al., 2010). A limitation of our study is that we performed all phases of the fear conditioning and extinction task during the single session in the scanner, which meant that we assessed extinction recall on the same day as extinction (after a rest period). Due to this, recall was based on one trial, but numerous studies have used one or two trials to assess recall (Milad et al., 2005, 2006, 2007; Hartley et al., 2011; Rabinak et al., 2013; Forcadell et al., 2017). It was a strength to be able to examine across-phase differences in brain activity, but it is possible that this is why we observed no significant neural differences between adults and adolescents during the re-extinction phase. Future studies can examine extinction learning vs. extinction recall vs. re-extinction in adolescents and adults in separate fMRI sessions and determine whether the length of time between those sessions change how extinction is processed at both the behavioral and neural level. Our study design, whereby extinction occurred immediately after conditioning, highlights a gap in the adolescent rodent literature, as no studies to date have reported whether adolescent extinction deficits occur when extinction is carried out immediately after conditioning. One study reported that in preadolescent rats, overall extinction recall was better following immediate extinction compared to delayed extinction, but the recall levels were not compared against any other age group (Kim and Richardson, 2009). In adult rodents, findings are mixed with some studies reporting better extinction recall while others reporting worse extinction recall when extinction is given immediately after conditioning (Maren and Chang, 2006; Myers et al., 2006; Monfils et al., 2009). Interestingly, a study in adult humans examined immediate vs. delayed extinction, and observed a small effect of immediate extinction leading to better extinction recall (Norrholm et al., 2008). Future research should assess whether the conditioning-extinction interval also influences extinction recall during adolescence.

## Conclusion

We have established that healthy human adolescents show different behavioral and neural responses to extinction recall as compared to adults. These findings may add to the growing evidence that adolescents are impaired in remembering extinction (Kim and Ganella, 2015). Importantly, our findings have relevance for understanding anxiety vulnerability and treatment in adolescents. There is clinical evidence that extinction-based therapy for anxiety disorders is less effective in adolescents compared to other ages (Southam-Gerow et al., 2001; Bodden et al., 2008). Our findings might suggest a neural basis for this finding, although future research is needed to examine whether and how the neural correlates of extinction recall in adolescents is related to anxiety and anxiety disorder treatment. In summary, our findings contribute to a more comprehensive understanding of the neural circuitry underlying extinction in adolescents, which may be critical for the development of age-specific treatments for anxiety disorders.

## Author contributions

DG, SW, and JK conceptualized and designed the study. DG acquired the data. DG, KD, EG, SW, and JK analyzed the data. DG, SW, and JK interpreted the data and wrote the paper. All authors were involved in the work for important intellectual content and in final approval of the version to be published. All authors agree to be accountable for all aspects of the work in ensuring that questions related to the accuracy or integrity of any part of the work are appropriately investigated and resolved.

### Conflict of interest statement

The authors declare that the research was conducted in the absence of any commercial or financial relationships that could be construed as a potential conflict of interest.
